# Effect of Different Expansive Agents on the Deformation Properties of Core Concrete in a Steel Tube with a Harsh Temperature History

**DOI:** 10.3390/ma16051780

**Published:** 2023-02-21

**Authors:** Anqun Lu, Wen Xu, Qianqian Wang, Rui Wang, Zhiyuan Ye

**Affiliations:** 1 State Key Laboratory of High Performance Civil Engineering Materials, Jiangsu Research Institute of Building Science, Nanjing 210008, China; 2 Research Institute of Jiangsu Sobute New Materials Co., Ltd., Nanjing 211103, China; 3 School of Materials Science and Engineering, Southeast University, Nanjing 211189, China; 4 College of Materials Science and Engineering, Nanjing Tech University, Nanjing 210009, China; 5 Lalin Railway Construction Headquarters of China Railway Corporation, Linzhi 860114, China

**Keywords:** CaO and MgO composite expansive agent, reaction time of MgO, temperature history, expansion properties

## Abstract

The shrinkage of core concrete during construction is the key reason for the separation of steel pipes and core concrete. Utilizing expansive agents during cement hydration is one of the main techniques to prevent voids between steel pipes and core concrete and increase the structural stability of concrete-filled steel tubes. The expansion and hydration properties of CaO, MgO, and CaO + MgO composite expansive agents in C60 concrete under variable temperature conditions were investigated. The effects of the calcium–magnesium ratio and magnesium oxide activity on deformation are the main parameters to consider when designing composite expansive agents. The results showed that the expansion effect of CaO expansive agents was predominant in the heating stage (from 20.0 °C to 72.0 °C at 3 °C/h), while there was no expansion in the cooling stage (from 72.0 °C to 30.0 °C at 3 °C/d, and then to 20.0 °C at 0.7 °C/h); the expansion deformation in the cooling stage was mainly caused by the MgO expansive agent. With the increase in the active reaction time of MgO, the hydration of MgO in the heating stage of concrete decreased, and the expansion of MgO in the cooling stage increased. During the cooling stage, 120 s MgO and 220 s MgO resulted in continuous expansion, and the expansion curve did not converge, while 65 s MgO reacted with water to form brucite in large amounts, leading to its lower expansion deformation during the later cooling process. In summary, the CaO and 220 s MgO composite expansive agent in the appropriate dosage is suitable for compensating for the shrinkage of concrete in the case of a fast high-temperature rise and slow cooling rate. This work will guide the application of different types of CaO-MgO composite expansive agents in concrete-filled steel tube structures under harsh environmental conditions.

## 1. Introduction

Concrete-filled steel tubes, as composite structures, can enhance the performance of concrete and steel. It has a higher bearing capacity, stability, and economic benefit when compared to the individual components; hence, it is frequently utilized in large bridge structures [1,2,3]. Materials scientists and structural engineers have made great progress on its wide application, and hundreds of concrete-filled steel tube arch bridges have been built or are under construction worldwide, which has resulted in a wide range of structural forms and significant progress in the building of long-span arch bridges [4,5,6,7]. However, in the construction of concrete-filled steel tubes, the problems of interface voids and debonding are common, affecting the synergy between them and endangering structural safety [8,9]. The debonding and voiding of concrete-filled steel tubes happen for a variety of reasons, including axial compression, shrinkage and creep of the concrete itself, and internal and exterior temperature variations caused by sunlight [10,11,12]. The interface void problem occurs once the construction period of most arch bridges is finished, indicating that the shrinkage of concrete during the construction period is the primary reason for voids [13]. The core concrete in the steel tube has a high strength grade in general, and it experiences a severe temperature drop during its early hydration period. Uneven internal and exterior deformations will cause the concrete surface to debond from the steel. In the later stage, the superposition of concrete temperature-drop-induced shrinkage and autogenous shrinkage will further aggravate the void problem. Therefore, controlling the shrinkage of concrete in concrete-filled steel tubes has emerged as an important topic [14].

Decreasing the shrinkage or causing the slight expansion of the concrete in the steel pipe could be crucial to preventing the separation of the steel pipe and concrete. Utilizing expansive agents during cement hydration is one of the main techniques to prevent voids between the steel pipe and core concrete and increase the structural stability of concrete-filled steel tubes [15]. In cement and concrete, three types of expansive agents are commonly used: sulfoaluminate hydrated ettringite [16,17,18], calcium oxide (CaO) hydrated calcium hydroxide [19,20], and magnesium oxide (MgO) hydrated magnesium hydroxide [21,22,23]. The classic calcium sulfoaluminate expansive agent has the disadvantages of high water demand and unstable hydration products at high temperatures, limiting its usage in high-strength concrete with a lower water–binder ratio [24,25]. The calcium oxide expansive agent is widely used in concrete-filled steel tube structures [26], but its hydration speed is too fast, the adjustability of the expansion process is poor, and its hydration is largely ineffective before the formation of the concrete slurry aggregate structure (that is, the plastic stage) [27]. According to previous studies [28,29], the compensatory impact of calcium oxide expansive agents on the cooling shrinkage and drying shrinkage of high-strength concrete is insignificant. Compared with the CaO expansive agent, the hydration products of the MgO expansive agent are more stable, and its expansion process can be much easier to control [30,31]. The MgO expansive agent has been widely used to compensate for the cooling shrinkage and autogenous shrinkage of hydraulic mass concrete [32,33]. Yao et al. [34] and Cai et al. [35] examined the deformation properties of a microexpansive concrete-filled steel tube with a MgO-based expansive agent at room temperature.

Since the temperature of concrete changes during the construction process, the performance of concrete at room temperature cannot reflect its application performance in the actual building. For example, for a steel tube filled with C50~C80 concrete with a large pipe diameter, the central temperature of core concrete can reach 50~60 °C. Due to heat dissipation to the environment, the concrete will undergo a rapid temperature drop after reaching the temperature peak. For example, during the construction of the Zangmu Bridge in Tibetan areas in China, the average temperature drop rate of the core concrete reached 3 °C/d after the temperature rise, which created a big challenge and required building non-void concrete-filled steel tubes. Thus, studying the effect of an expansive agent exposed to the actual temperature history is more valuable for engineering applications.

Furthermore, utilizing a single type of expansive agent to compensate for the shrinkage deformation of concrete in different stages is not sufficient. Liu et al. [36] designed a magnesium oxide composite expansion agent composed of specific proportions of high-activity MgO, low-activity MgO, and CaO at 20 °C, which effectively eliminated the early autogenous shrinkage of high-performance concrete and markedly inhibited its drying shrinkage. Yu et al. [37] also explored a type of multisource expansive agent mixed with different proportions of calcium oxide, calcium sulfoaluminate, and MgO in high-strength concrete, which revealed that the multisource expansive agent can effectively compensate for the drying shrinkage of high-strength concrete at normal temperatures in the later stage. Thus, previous research on cement-based materials mixed with CaO and MgO composite expansive agents has mainly focused on strength and deformation development at constant temperatures. Recently, Zhao et al. [38] studied the effect of a blended MgO-CaO expansive agent on the hydration of cement paste at an early age by using low-field nuclear magnetic resonance technology, and they found that the curing temperature has a great impact on the hydration of the cement paste mixed with the MgO-CaO blended expansive agent. However, the influence of the activity of MgO in the CaO and MgO composite expansive agent on the deformation properties has not been studied. Additionally, there are few studies on the influence of the calcium-to-magnesium ratio of CaO and MgO composite expansive agents on the deformation of concrete when they are under variable temperature conditions during the construction process.

This work investigates the deformation performance of C60 concrete mixed with different contents of a CaO expansive agent, MgO expansive agent, and CaO-MgO composite expansive agents under the condition of a simulated temperature history. The temperature variation process used in this work was first monitored for a part of the core concrete in the steel tubes of the Zangmu Bridge in Tibet in China. This temperature history could be suitable for simulating the deformation performance of concrete under the typically closed condition of the harsh plateau environment. Afterward, a reasonable amount of a calcium oxide and magnesium oxide composite expansive agent was mixed with concrete and poured into tubes. The effect of the CaO-MgO ratio of the composite expansive agent on the concrete was investigated by testing its deformation performance by using an SBT-CDMI wireless monitoring system, and the activity time of MgO on the deformation was considered. Finally, the hydration products and microstructures of cement pastes with different types of expansive agents were also studied. These findings will guide the use of CaO-MgO composite expansive agents in concrete-filled steel tubes and other non-shrink concrete.

## 2. Materials and Methods

### 2.1. Materials

The cement used in this study was ordinary Portland cement 42.5, which was produced by Conch Cement Plant, and conformed to the Chinese standard GB175-2007 [39]. Fly ash was Class Ⅱ fly ash from the Huaneng Power Plant. Natural river sand with a fineness modulus of 2.60 was used. The aggregate was basalt gravel. The particle size of small stones was 5–20 mm. The particle size of medium stones was 20–40 mm. The superplasticizer was PCA polycarboxylic acid superplasticizer produced by Jiangsu Sobute New Materials Co., Ltd., Nanjing, China.

Three MgO expansive agents with different active reaction times (65 s, 120 s, and 220 s) were prepared by calcining magnesite in a suspension kiln. The 65 s, 120 s, and 220 s MgO expansive agents were calcined from magnesite powder (particle size ≤ 160 μm) at 800 °C, 950 °C, and 1050 °C, respectively, in a suspension kiln. The citric acid method was used to determine the active reaction time of the MgO expansive agent [40]. The active reaction time was determined by using 1.70 ± 0.1 g of the MgO expansive agent to completely neutralize 200 mL of a 0.07 mol/L citric acid solution at 30 ± 1 °C, which was used as a measure for evaluating the activity of the MgO expansive agent. Obviously, a shorter active reaction time means higher activity [30]. The CaO expansive agent was prepared by calcining limestone and a mineralizer in a rotary kiln at 1350 °C. Both the MgO expansive agent and CaO expansive agent were provided by Jiangsu Sobute New Materials Co., Ltd., Nanjing, China. The chemical components of cement, the MgO expansive agent, and the CaO expansive agent are demonstrated in Table 1.

**Figure 1:**
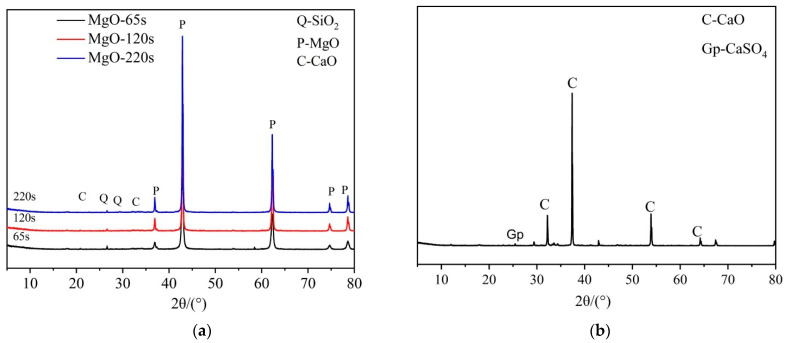
XRD patterns of expansive agents: (**a**) MgO expansive agents and (**b**) CaO expansive agents.

The XRD patterns of MgO expansive agents and CaO expansive agents are shown in Figure 1. The 65 s MgO, 120 s MgO, and 220 s MgO expansive agents have MgO as the main mineral and contain small amounts of SiO_2_ and CaO. The primary mineral of the CaO expansive agent is CaO, containing a small amount of CaSO_4_.

### 2.2. Experiments

#### 2.2.1. Mix Design of Concrete

Table 2 displays the concrete proportion for the C60 strength grade. MgO and CaO expansive agents were mixed internally. The slump of the concrete was controlled by the superplasticizer and other admixtures at 180–200 mm with 3.0–5.0% air content. The table also shows the amount of fly ash in the design of C60 concrete. Single CaO expansive agents, single MgO expansive agents, and compound expansive agents in C60 concrete were added to the mixture. It can be seen from the table that 6% CaO + 2% 65 s MgO indicates that the 6% calcium oxide expansive agent and the 2% magnesium oxide expansive agent with an active reaction time of 65 s are mixed in the concrete.

Replacing a portion of the cement with fly ash can reduce the amount of heat released in concrete, the adiabatic temperature rise in concrete, and the autogenous shrinkage of concrete, which decreases the risk of concrete cracking, particularly in concrete containing a large amount of fly ash. Therefore, fly ash was used in this design for its application in concrete-filled steel tubes. The added content of MgO was selected according to T/CECS 10082—2020 Calcium and Magnesium Oxides Based Expansive Agent for Concrete [41].

#### 2.2.2. Test of Deformation Performance of C60 Concrete under Variable Temperature Conditions

**Figure 2:**
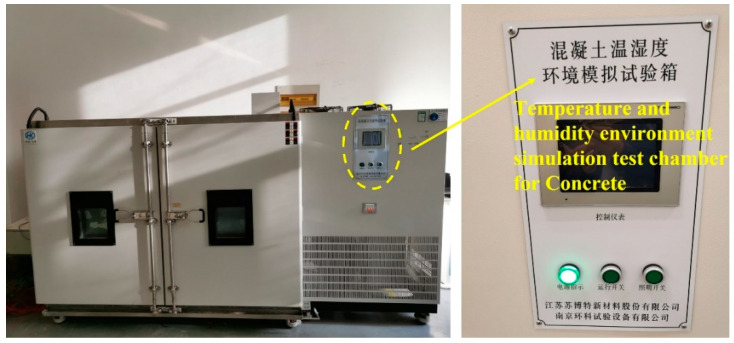
Temperature and humidity environment simulation test chamber for concrete.

**Figure 3:**
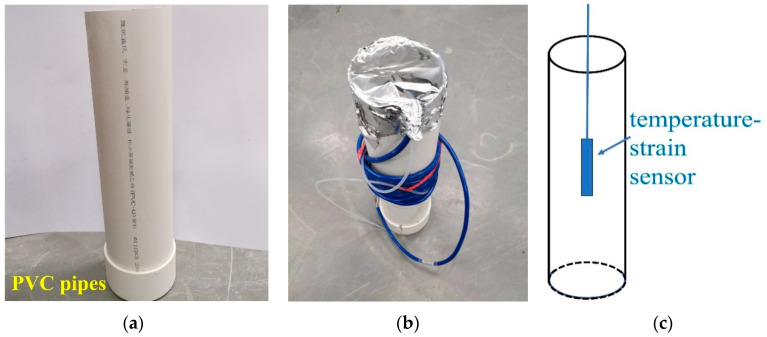
PVC pipe and PVC pipe filled with concrete and temperature–strain sensor embedded in the central part: (**a**) PVC pipe, (**b**) PVC pipe containing concrete, and (**c**) schematic diagram.

**Figure 4:**
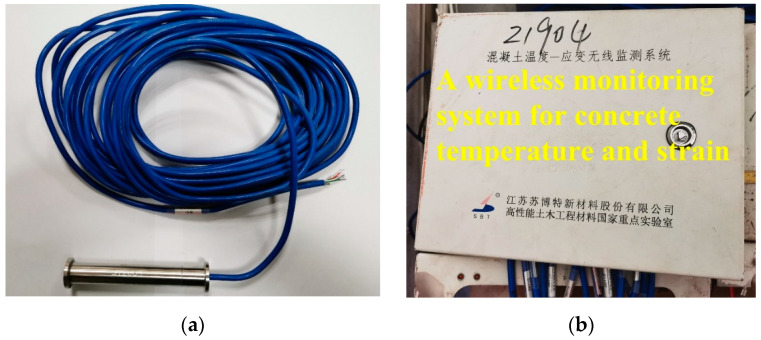
Monitoring by SBT-CDMI wireless monitoring system for concrete temperature and strain: (**a**) sensor and (**b**) data receiving system.

**Figure 5:**
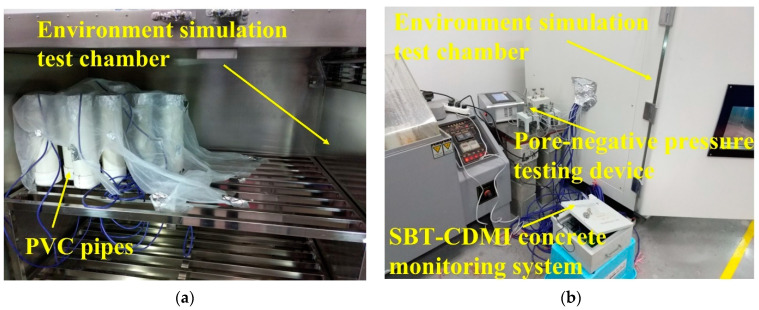
Concrete deformation and temperature monitoring test: (**a**) test molds and devices and (**b**) data receiving system.

The test was conducted using a temperature and humidity environment simulation test chamber for concrete made by Nanjing Huanke Testing Equipment Co., Ltd., Nanjing, China., to simulate the temperature history of a C60 concrete-filled steel tube. The temperature and humidity environment simulation test chamber for concrete is shown in Figure 2. First, fresh C60 concrete was poured into polyvinyl chloride (PVC for short) pipes (Φ160 mm × 400 mm, shown in Figure 3). Then, a temperature–strain sensor was embedded in the center of the PVC pipe that had been filled with concrete (shown in Figure 4). The upper part of the PVC pipe was sealed with tin foil. Finally, the PVC pipes were put into the test chamber to simulate the environment (shown in Figure 5). The experiments used a pore water pressure testing device to measure the development of pore water pressure in concrete to determine the setting time [42]. An SBT-CDMI wireless monitoring system for the concrete temperature and strain was used to monitor the concrete’s deformation and temperature history. The wireless monitoring system and the pore water pressure testing device were provided by Jiangsu Sobute New Materials Co. Ltd., Nanjing, China.

The C60 concrete in the PVC pipe was in a state of absolute humidity. The concrete deformation recorded by the wireless monitoring system was mainly caused by autogenous volume deformation and temperature deformation. The temperature history of the concrete was mainly controlled by the environmental temperature change in the test chamber. Firstly, the test chamber was started, and the initial temperature inside the chamber was controlled at (20.0 ± 1.0) °C. After 20 h at a constant temperature, the test chamber was heated up to (72.0 ± 1.0) °C at 3 °C/h, then cooled down to (30.0 ± 1.0) °C at 3 °C/d, and then cooled down to (20.0 ± 1.0) °C at 0.7 °C/h. After the temperature fell to (20.0 ± 1.0) °C, the test chamber was turned off. The temperature of concrete varied with the external environment. The temperature variation process used in this work was first monitored for a part of the core concrete in the steel tubes of the Zangmu Bridge in Tibet in China. Its typical temperature history can be used to simulate the deformation performance of concrete embedded in large-diameter tubes under the closed condition of the harsh plateau environment.

#### 2.2.3. Hydration Heat

Samples were placed at a constant temperature of 20 °C about 24 h before the experiment. Then, 10.0 g of the expansive agent was weighed and put into an ampoule bottle, and 10 mL of water was injected into the bottle with a syringe. Then, the paste was stirred quickly and evenly and put into the test channel of a TAM AIR isothermal calorimeter. The hydration and cumulative heat release rates were tested for 3 consecutive days.

#### 2.2.4. Measurement of Hydration Degree of MgO Expansion Agent under Variable Temperature Conditions

**Figure 6:**
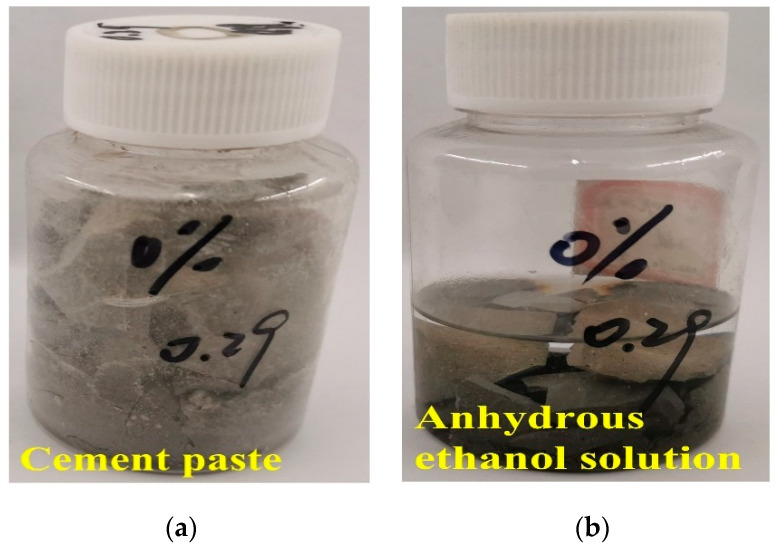
Cementitious material paste specimen poured into a 100mL plastic test tube: (**a**) cement paste in a plastic test tube and (**b**) cement paste sample dehydrated in anhydrous ethanol solution.

To prevent the influence of components such as sand and gravel aggregate on the analysis of experimental results, the experiment was conducted with a cementitious material paste specimen. The expansive agent replaces the fly ash in an equal amount and does not replace the cement. The cement paste specimen was made according to a water–binder ratio of 0.29. After the paste sample was stirred evenly, it was poured into a 100 mL plastic test tube (shown in Figure 6). The plastic test tube and the above PVC pipes filled with concrete were put into the environmental simulation test chamber. After the variable-temperature experiment, the plastic test tube was taken out. The sample was put into an agate mortar, mixed, and ground with an appropriate amount of absolute ethanol until it passed through an 80 μm square sieve. The test instrument was a D8 Advance X-ray diffractometer manufactured by Bruker Company in Ettlingen, Germany, with a voltage of 40 kV, current of 200 mA, and scanning angle range of 5°~70°. The quantitative analysis of Rietveld full-spectrum fitting was performed with Jade 6 software.

#### 2.2.5. SEM Analyses of Cement Paste Mixed with Expansive Agent

The micromorphology of cement paste with the expansive agent was characterized by SEM. The samples were processed as follows: cutting the two ends of the hardened paste in the plastic bottle to 5 mm, crushing the rest into small pieces, putting them into anhydrous ethanol for dehydration for 24 h (shown in Figure 6), drying them to constant weight, and sealing them for storage. A Quanta 250 field-emission scanning microscope manufactured by FEI Company in Hillsboro, OR, USA, was used to observe the micromorphology of a fresh fracture in the slurry. The fracture surface was presprayed with gold film.

## 3. Results and Discussion

### 3.1. Expansion Properties of CaO Expansive Agents in C60 Concrete under Variable Temperature Conditions

**Figure 7:**
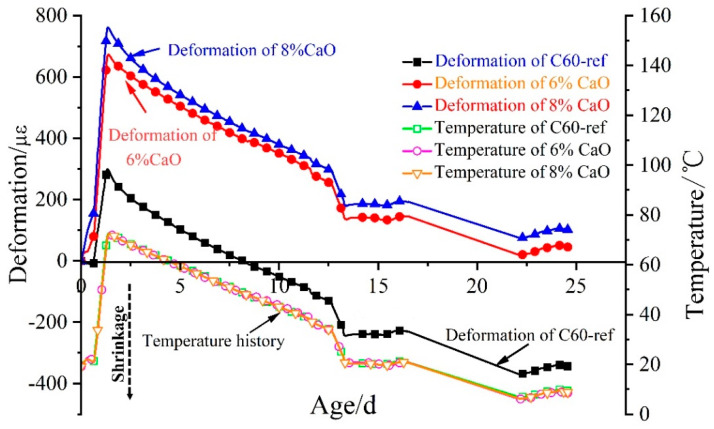
Deformation and temperature history of C60 concrete with 0%, 6%, and 8% CaO expansive agents.

The autogenous volume deformation and temperature history of C60 concrete with 0%, 6%, and 8% CaO expansive agents under simulated variable temperature conditions are shown in Figure 7. The experiment used a pore water pressure testing device to measure the development of pore water pressure in concrete to determine the setting time. The time was taken as the initial setting time when the pore water pressure in concrete reached 10 kPa [42]. With the initial setting time as the zero point, the C60-ref reference concrete in the heating stage underwent 298 με expansion deformation, and the temperature rose to 52.5 °C. Moreover, C60 concrete with the 6% CaO expansive agent in the heating stage gave rise to 672 με expansion deformation, and the temperature rose to 53.1 °C. In addition, C60 concrete with the 8% CaO expansive agent in the heating stage triggered 761 με expansion deformation, and the temperature rose to 53.4 °C. C60 concrete containing the CaO expansive agent was still in an expansion state at 24.5 days after the initial setting time under variable temperature conditions.

**Figure 8:**
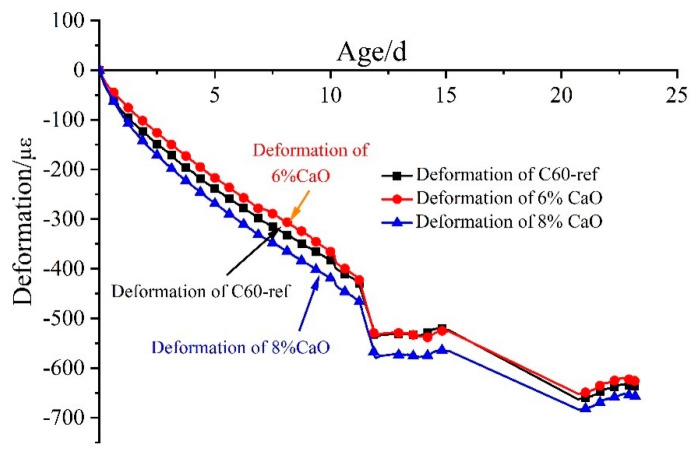
Deformation process of C60 concrete with 0%, 6%, and 8% CaO expansive agents in cooling stage.

The deformation process of C60 concrete with 0%, 6%, and 8% CaO expansive agents in the cooling stage is depicted in Figure 8. The shrinkage of C60-ref reference concrete was 637 με at 23 d of age in the cooling stage, while the shrinkage of C60 concrete with the 6% CaO expansive agent was 626 με. Furthermore, the shrinkage of C60 concrete with the 8% CaO expansive agent was 657 με. Among them, the shrinkage of C60 concrete with the 6% CaO expansive agent in the cooling phase was similar to that of C60-ref concrete, while the shrinkage of concrete with the 8% CaO expansive agent in the cooling phase was slightly larger than that of C60-ref concrete.

The hydration of the CaO expansive agent in concrete to form Ca(OH)_2_ crystals will result in the volume expansion of concrete, especially with higher amounts of the CaO expansive agent. Details are provided in Section 3.7.

**Figure 9:**
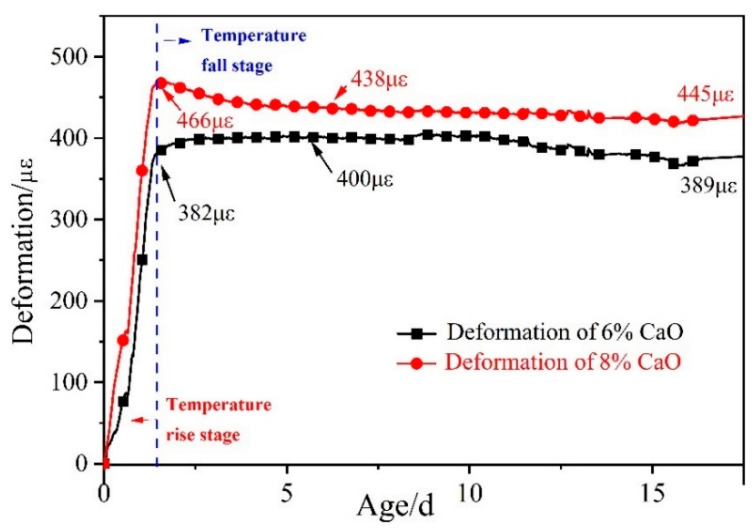
Expansion performance of 6% and 8% CaO expansive agents after deducting deformation of C60-ref.

Figure 9 illustrates the expansion deformation of 6% and 8% CaO expansive agents and the base concrete (C60-ref)’s net deformation effects, with the initial setting time as the zero point. The expansion of the CaO expansive agent was significant during the heating stage of the concrete. The concrete reached the temperature peak at 1.46 d after the initial setting time, and the 6% CaO expansive agent generated 382 με expansion deformation. Subsequently, the 6% CaO expansive agent generated 18 με expansion deformation during the cooling stage from 1.46 d to 3.00 d. At 24.5 d, the expansion produced by the 6% CaO expansive agent was 389 με. During the cooling stage from 1.46 d to 24.5 d, the expansion of the 6% CaO expansive agent increased by only 7 με. The concrete with the 8% CaO expansive agent reached the temperature peak at 1.46 d after the initial setting time, and the 8% CaO expansive agent generated 466 με expansion deformation. During the cooling stage from 1.46 d to 3.00 d, the expansion of the 8% CaO expansive agent was reduced by 17 με. From the initial setting time to 24.5 d, the 8% CaO expansive agent generated 445 με expansion deformation. During 24.5 days after the initial setting time, the expansion of the 6% CaO expansive agent in the heating stage accounted for 98.2% of the total expansion. The expansion of the 8% CaO expansive agent in the heating stage decreased by 4.5% at 24.5 d.

As further demonstrated by the above experimental results, the expansion effects of 6% and 8% CaO expansive agents were predominantly reflected in the heating stage, while there was no expansion in the cooling stage. The CaO expansive agent might have been consumed by hydration in the early high-temperature heating stage. So, there was almost no expansion in the cooling stage. With the increase in the amount of the CaO expansive agent, the expansion deformation of concrete in the heating stage increased, and consequently, the internal expansion stress of concrete increased. C60 concrete with the 8% CaO expansive agent triggered higher expansion stress in the heating stage, which led to the augmentation of concrete creep in the cooling stage. Accordingly, the expansion amount with the 8% CaO expansive agent appeared to shrink back in the cooling stage.

### 3.2. Expansion Properties of MgO Expansive Agents in C60 Concrete under Variable Temperature Conditions

**Figure 10:**
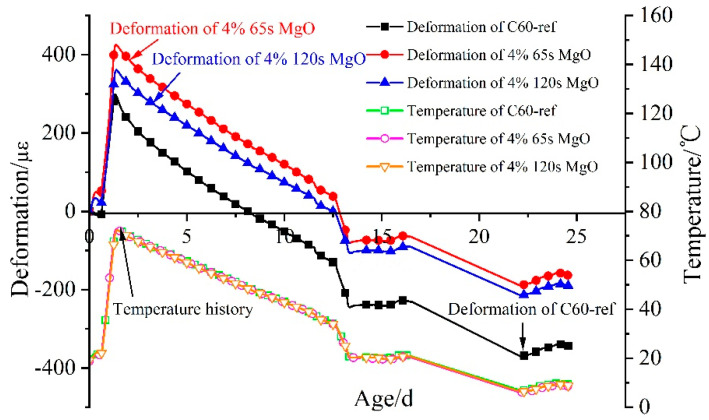
Deformation and temperature history of C60 concrete with 0%, 4% 65 s, and 4% 120 s MgO expansive agents.

As revealed by the above experimental findings, the distinction in the expansion effects of CaO expansive agents at distinct dosing levels was primarily reflected in the heating stage. For C60 concrete structures, CaO expansive agents could only compensate for the early autogenous shrinkage of concrete. The autogenous volume deformation and temperature history of C60 concrete with 0%, 4% 65 s MgO, and 4% 120 s MgO expansive agents subjected to a simulated temperature history are displayed in Figure 10. C60 concrete with the 4% MgO expansive agent was in a state of shrinkage at 24.5 d after the initial setting time. With the initial setting time as the zero point, C60 concrete with the 4% 65 s MgO expansive agent in the heating stage resulted in 422 με expansion deformation. Furthermore, C60 concrete with the 4% 120 s MgO expansive agent gave rise to 359 με expansion deformation. The reaction rate of the highly active MgO (65 s) expansive agent was faster in the heating stage of this temperature history.

**Figure 11:**
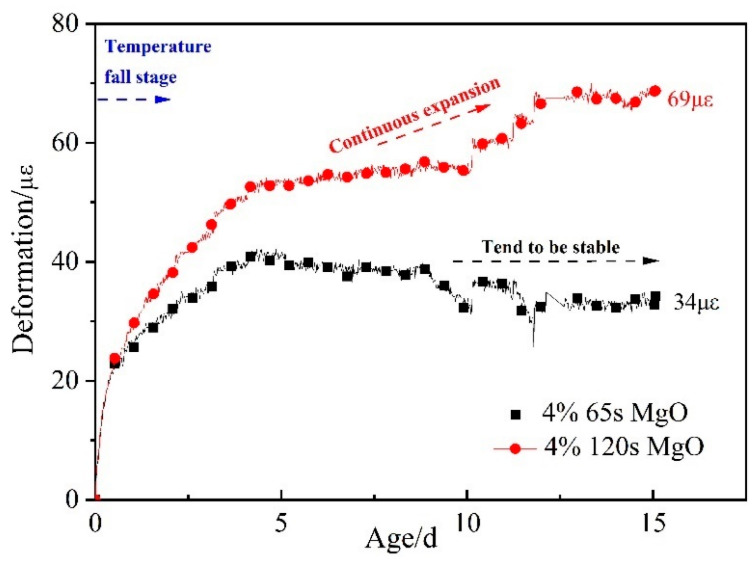
Expansion performance of 4% 65 s MgO and 4% 120 s MgO expansive agents in cooling stage after deducting deformation of C60-ref.

The expansion curves of 4% 65 s MgO and 4% 120 s MgO expansive agents in the cooling stage are shown in Figure 11, after deducting the deformation of C60-ref concrete and temperature effects. The deformation of the 4% 120 s MgO expansive agent continued to increase in the cooling stage. The 4% 65 s MgO expansive agent can also produce expansion deformation in the cooling stage. In comparison with the 65 s MgO expansive agent, the 120 s MgO expansive agent triggered continuous expansion and generated 69 με expansion deformation at 15 d in the cooling stage. As the active reaction time rises, the hydration of MgO in the heating process of concrete decreases, and the expansion in the cooling phase increases.

As demonstrated by the above experimental findings, in this temperature history, CaO expansive agents can only compensate for the early concrete shrinkage and store early expansion stress and have no expansion compensation effect in the cooling stage. If CaO expansive agents are employed to increase the amount of concrete expansion, there would be an increment in concrete creep during the cooling stage. Using the single admixtures of MgO expansive agents, C60 concrete with the 4% 65 s MgO or 4% 120 s MgO expansive agent was in a shrinkage state at 24.5 d after the initial setting time and had not reached the expansion or non-shrinkage state. If the experiment raises the amount of MgO to realize the objective of non-shrinkage, the workability of concrete will be seriously decreased, leading to the formation of cavities between the steel pipe and concrete. The experiments of single MgO expansive agents also show that with this temperature history, the activity reaction time of MgO increases, the hydration of MgO in the concrete heating stage decreases, and the expansion of MgO in the cooling stage rises.

### 3.3. Expansion Properties of Expansive Agents of 6% CaO and 2% MgO with Different Active Times in C60 Concrete under Variable Temperature Conditions

Whether the delayed expansion of MgO could be employed to compensate for the shrinkage of concrete during the cooling stage and realize the compensation for shrinkage deformation in concrete remains for the complete process to be determined. For the sake of selecting suitable CaO and MgO composite expansive agents to compensate for the shrinkage of C60 concrete in the whole process, the expansion deformation laws of expansive agents with 2% MgO (active time of 65 s, 120 s, or 220 s) and 6% CaO composite were compared and analyzed.

**Figure 12:**
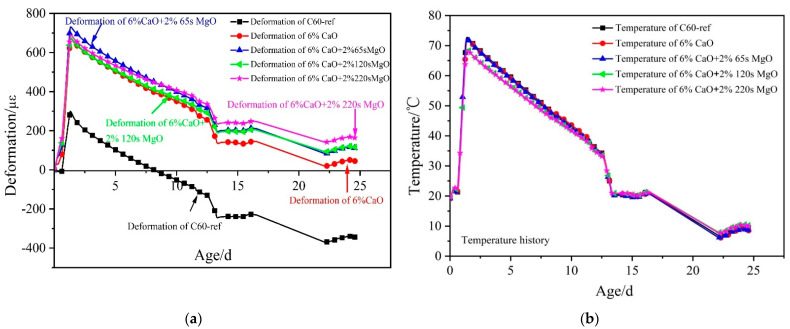
Deformation and temperature history of C60 concrete with 0%, 6% CaO, and 6% CaO + 2% MgO expansive agents: (**a**) deformation history and (**b**) temperature history.

The volume deformation and temperature history of C60 concrete with 0%, 6% CaO, and 6% CaO + 2% MgO expansive agents under simulated variable temperature are depicted in Figure 12. Compared with the base concrete (C60-ref), the expansion deformation of C60 concrete with 6% CaO + 2% MgO increased in the heating stage, while the temperature development history of each proportion of concrete was similar. As a consequence, it could be assumed that the increment in expansion was due to the hydration of expansive agents. With the initial setting time as the zero point, C60 concrete with 6% CaO + 2% 65 s MgO in the heating stage was the highest with 731 με expansion deformation, and the temperature rose to 53.0 °C. Moreover, C60 concrete with the 6% CaO + 2% 120 s MgO expansive agent in the heating stage gave rise to 675 με expansion deformation, and the temperature rose to 48.9 °C. Additionally, C60 concrete with the 6% CaO + 2% 220 s MgO expansive agent in the heating stage triggered 689 με expansion deformation, and the temperature rose to 48.8 °C.

**Figure 13:**
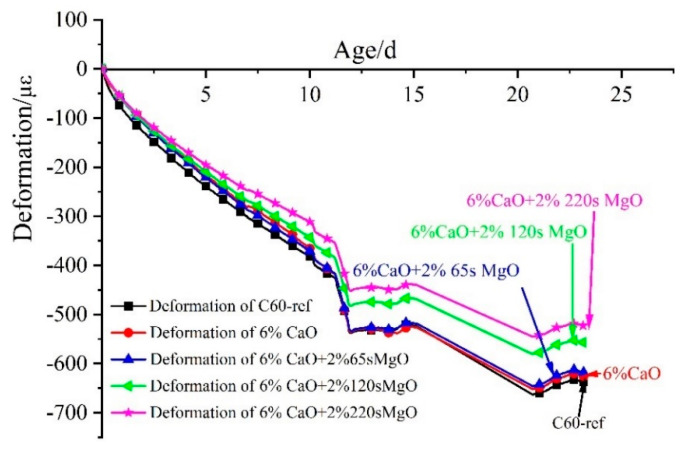
Deformation process of C60 with 0%, 6% CaO, and 6% CaO + 2% MgO expansive agents in cooling stage.

The deformation process of C60 with 0%, 6% CaO, and 6% CaO + 2% MgO expansive agents in the cooling stage is depicted in Figure 13. The shrinkage of C60 concrete with CaO + 2% MgO composite expansive agents was reduced compared to the base concrete (C60-ref) during the cooling stage. The expansive agents of CaO are consumed by hydration in the early heating process, which demonstrates that the expansion in the cooling phase originates from the hydration of MgO, which could undergo expansion during an appropriate cooling course to compensate for the shrinkage. During the cooling stage, deducting the influence of the shrinkage of C60-ref concrete, the 6% CaO + 2% 65 s MgO expansive agent generated 20 με expansion deformation. In addition, the 6% CaO + 2% 120 s MgO expansive agent resulted in 81 με expansion deformation. Most significantly, the 6% CaO + 2% 220 s MgO expansive agent triggered 115 με expansion deformation, indicating that 220 s MgO exhibits the best compensation effect in the cooling stage.

**Figure 14:**
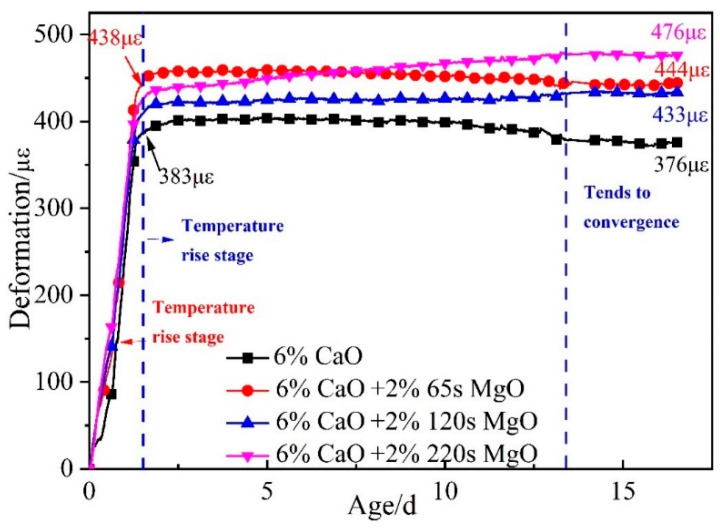
Expansion performance of 6% CaO and 6% CaO + 2% MgO expansive agents after deducting the deformation of C60-ref.

Figure 14 illustrates the expansion deformation of the 6% CaO expansive agent and 6% CaO + 2% MgO expansive agents and the base concrete (C60-ref)’s net deformation and temperature effects, with the initial setting time as the zero point. The expansion effect of CaO expansive agents was predominantly reflected in the heating stage. The expansion deformation during the cooling stage was mainly caused by the MgO expansive agent. Using 120 s MgO and 220 s MgO resulted in continuous expansion during the cooling stage, and the expansion curve did not converge. Using the 220 s MgO expansive agent produced more expansion than 120 s MgO, whereas 65 s MgO did not produce significant expansion deformation in the cooling phase (shown in Figure 14). During the heating process of the concrete, the majority of 2% 65 s MgO may have reacted with water to form brucite, leading to the observed phenomenon. Thus, with the increase in the active reaction time of MgO, the hydration of MgO in the heating stage of concrete decreased, and the expansion of MgO in the cooling stage increased.

### 3.4. Expansion Properties of Expansive Agents of 6% CaO and 65 s MgO in Different Proportions in C60 Concrete under Variable Temperature Conditions

**Figure 15:**
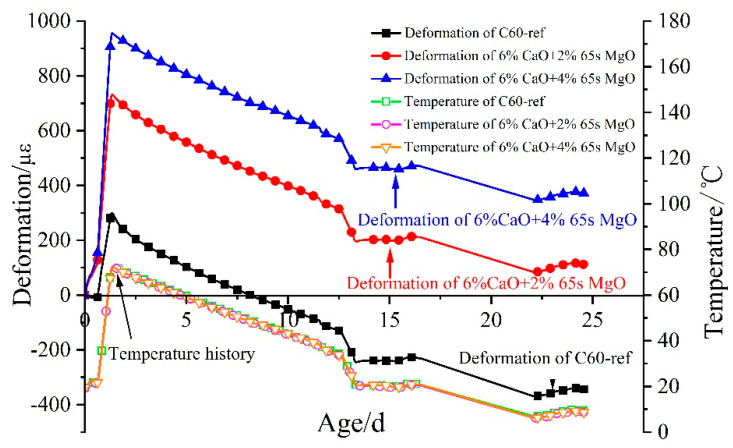
Deformation and temperature history of C60 concrete with 0%, 6% CaO + 2% 65 s MgO, 6% CaO + 4% 65 s MgO expansive agents.

The autogenous volume deformation and temperature history of C60 concrete with 0%, 6% CaO + 2% 65 s MgO, and 6% CaO + 4% 65 s MgO expansive agents under simulated variable temperature conditions are shown in Figure 15. With the initial setting time as the zero point, C60 concrete with 6% CaO + 2% 65 s MgO in the heating stage gave rise to 729 με expansion deformation, and the temperature rise was 52.7 °C. Moreover, C60 concrete with 6% CaO + 4% 65 s MgO in the heating stage triggered 953 με expansion deformation, and the temperature rise was 53.0 °C. With the increase in MgO content, the expansion of concrete in the heating stage increases.

**Figure 16:**
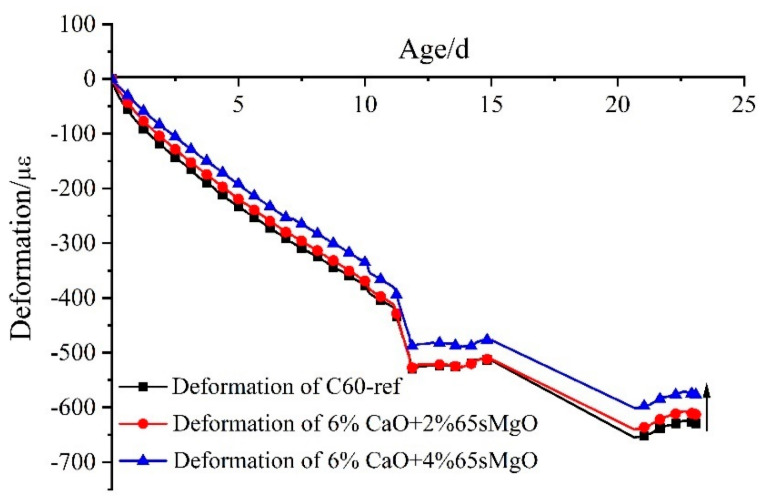
Deformation process of C60 with 6% CaO and 6% CaO + 4% MgO expansive agent at cooling stage.

The deformation process of C60 concrete with 0%, 6% CaO + 2% 65 s MgO, and 6% CaO + 4% 65 s MgO expansive agents at in cooling stage is depicted in Figure 16. With the increase in MgO content, the shrinkage of concrete in the cooling stage decreases. The shrinkage of C60 concrete with 6% CaO + 4% 65 s MgO decreased by 35 με at 23 d compared with C60 concrete with 6% CaO + 2% 65 s MgO.

**Figure 17:**
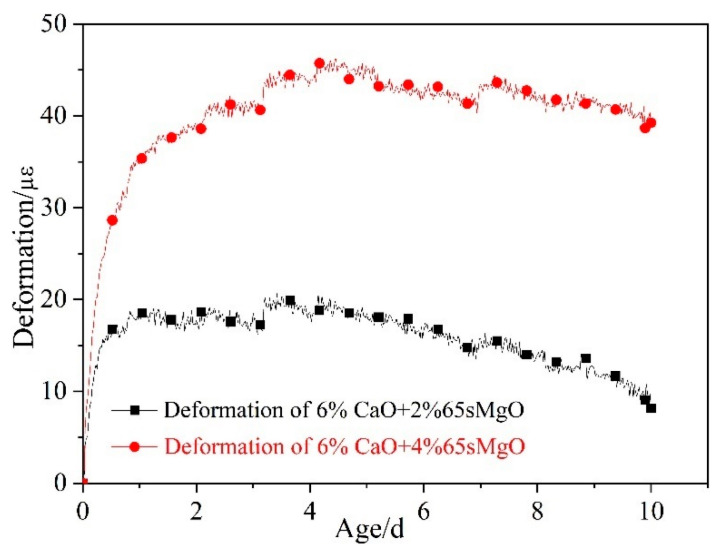
Expansion performance of 6% CaO + 2% MgO and 6% CaO + 4% MgO expansive agents in cooling stage after deducting deformation of C60-ref.

Figure 17 illustrates the expansion deformation of 6% CaO + 2% 65 s MgO and 6% CaO + 4% 65 s MgO expansive agents and the base concrete (C60-ref)’s net deformation and temperature effects, with the initial setting time as the zero point. The expansion of MgO in the cooling stage increased with the increment in 65 s MgO content. In comparison to 2% 65 s MgO, 4% 65 s MgO continued to expand in the cooling stage of C60 concrete, which demonstrates that expansion can also occur in the cooling stage as the amount of high-activity MgO increases.

As revealed by the experimental results, the CaO expansive agent has the advantages of a fast hydration rate, large expansion deformation, and a certain amount of expansion stress in concrete in the heating stage. In the cooling stage, concrete-filled steel tubes can achieve a microexpansion or non-shrinkage state by using the delayed expansion property of the MgO expansive agent. The hydration rate of longer-sintered MgO is higher. For the temperature history used, 2% 65 s MgO did not show significant expansion in the cooling stage, while expansion compensation could also be produced in the cooling stage when 65 s MgO expansive agent doping was increased to 4.0%. Under these temperature conditions, 220 s MgO with low activity demonstrated superior compensation for the cooling shrinkage. The expansion performance of 220 s MgO compounded with CaO and MgO composite expansive agents demonstrated strong temperature sensitivity, which is suitable for compensating for the shrinkage of C60 structural concrete with a high-temperature rise and slow cooling rate.

### 3.5. Isothermal Calorimetry of Different Expansive Agents

**Figure 18:**
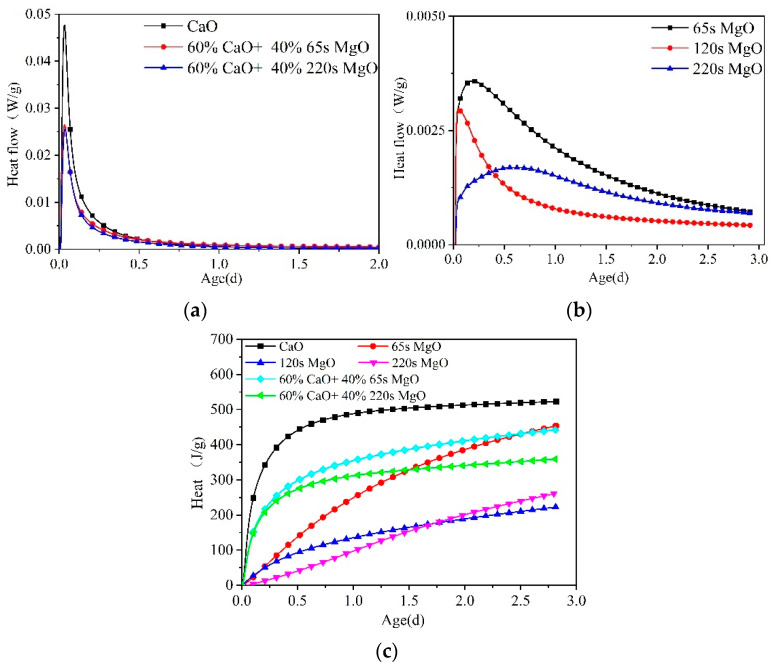
Hydration heat liberation of distinct expansive agents in pure water (paste) at 20 °C: (**a**) heat flow of CaO and CaO + MgO, (**b**) heat flow of MgO, and (**c**) total heat release of the expansive agent.

The exothermic courses of hydration of distinct expansive agents in pure water are demonstrated in Figure 18. At a constant temperature of 20 °C, the hydration rates of CaO expansive agents were faster. Furthermore, the accumulated exothermic heat was stabilized within 2 days. The 65 s MgO, 120 s MgO, and 220 s MgO expansive agents exhibited continuous augmentation in the accumulated exotherm over 2 days and yet did not show convergence, and the exothermic rate of hydration was significantly lower than that of CaO expansive agents. Additionally, the exotherm rate of hydration of 65 s MgO was significantly faster than that of 120 s and 220 s MgO. For CaO expansive agents in 20 °C water, the hydration reaction rates are fast, with the basic reaction complete in 2 days. In line with Arrhenius’s law, the reaction rate of CaO and MgO expansive agents must increase exponentially when the temperature rises. It is deduced that the CaO expansive agents completely reacted in the heating stage of this temperature history of C60 core concrete in the steel tube arch.

### 3.6. Hydration Degree of MgO Expansive Agent in Cement Paste under Variable Temperature Conditions

**Figure 19:**
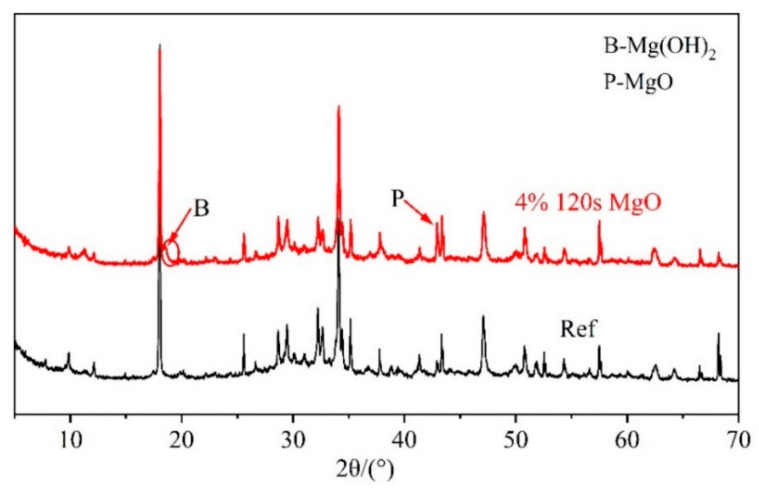
Comparison of XRD patterns of cement pastes with and without MgO expansive agent.

After being cured under variable temperature conditions, as shown in Figure 7, the XRD patterns of the cement paste (4% MgO, 16% fly ash, 80% cement, and water–binder ratio of 0.29) mixed with the 4% 120 s MgO expansive agent and the cement sample without an expansive agent (20% fly ash, 80% cement, and water–binder ratio of 0.29) were obtained and are shown in Figure 19. It can be seen that at 24.5 d after the initial setting time, the cement paste with the 4% 120 s MgO expansive agent contained MgO and Mg(OH)_2_ minerals. Table 3 shows the hydration degree of MgO in cement pastes with 6% CaO + 4% 65 s MgO, 4% 65 s MgO, and 4% 120 s MgO expansive agents at variable temperatures. At the same age, the hydration degree of 65 s MgO was higher than that of the 120 s MgO expansive agent. This shows that highly active MgO has higher hydration activity and a higher hydration rate. Whether the CaO expansive agent was added to the cement paste had little effect on the hydration degree of MgO. Under this variable temperature condition, more than 85% of the 65 s MgO expansive agent was almost completely hydrated. About 30% of MgO in the cement paste mixed with the 4% 120 s MgO expansive agent was not hydrated. The hydration degree of the MgO expansive agent is consistent with the change rule of the expansion amount of the MgO expansive agent (shown in Figure 10). The MgO expansive agent with high activity (65 s MgO) undergoes a large expansion in the early stage and a small expansion in the later stage. The MgO expansive agents with low activity (120 s MgO and 220 s MgO) undergo a small expansion in the early stage and a large expansion in the later stage.

### 3.7. SEM Analyses of Cement Paste Mixed with Expansion Agent

**Figure 20:**
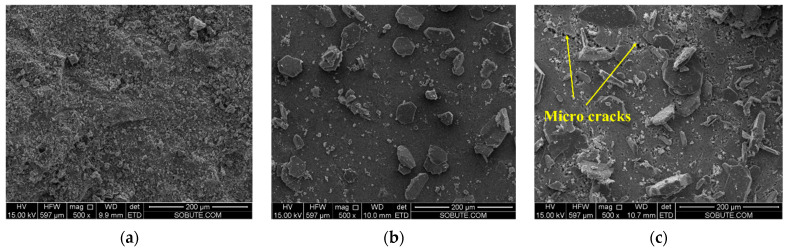
Morphologies of cement pastes with 0%, 4%, and 6% CaO expansive agents: (**a**) 0% CaO expansive agent, (**b**) 4% CaO expansive agent, and (**c**) 6% CaO expansive agent.

After being cured under variable temperature conditions, as shown in Figure 7, the morphologies of cement pastes with 0%, 4%, and 6% CaO expansive agents were observed by SEM and are shown in Figure 20. A large number of hexagonal plate Ca(OH)_2_ crystals appeared in the cement paste after adding the CaO expansive agent. As the content of the CaO expansive agent increased, the content of Ca(OH)_2_ generated by hydration also increased. As can be seen in Figure 20c, microcracks appeared in the cement paste with the 6% CaO expansive agent. The formation of microcracks may be caused by the volume expansion of Ca(OH)_2_ generated by the hydration of the CaO expansive agent. Microcracks were not observed in the cement paste containing the 4% CaO expansive agent. The reason for this could be that the crystallization pressure of Ca(OH)_2_ crystals generated by the 4% CaO expansive agent in the cement paste does not exceed the tensile strength of the cement paste [43].

**Figure 21:**
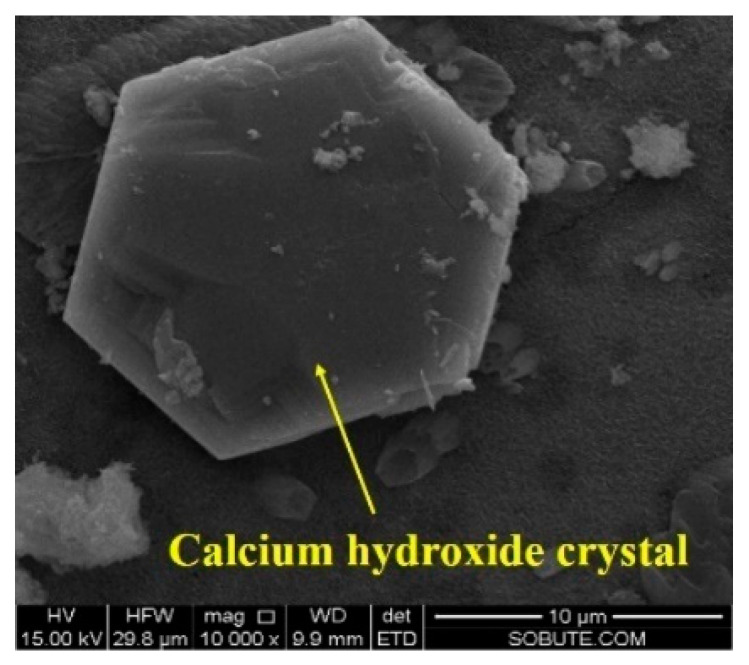
Ca(OH)_2_ with hexagonal plate structure formed by hydration of CaO expansive agent (×10,000).

**Figure 22:**
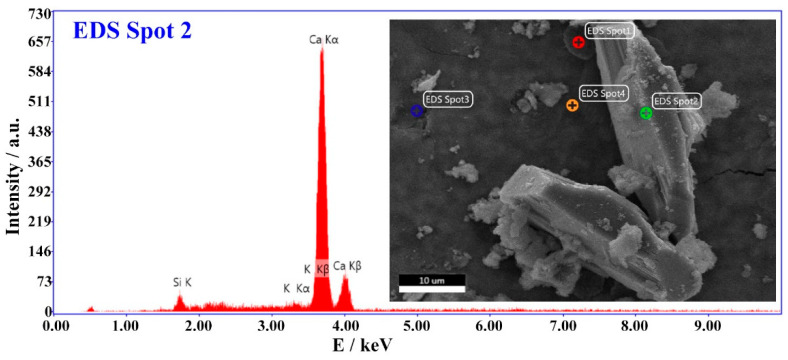
The energy spectrum test results of EDS Spot 2 in the microscopic picture of cement paste with 6% CaO expansive agent.

Figure 21 shows Ca(OH)_2_ with a hexagonal plate structure formed by the hydration of the CaO expansive agent. The energy spectrum test results of EDS Spot 2 in the microscopic picture of the cement paste with the 6% CaO expansive agent show that the hexagonal plate material consists of Ca(OH)_2_ crystals [44] (shown in Figure 22).

**Figure 23:**
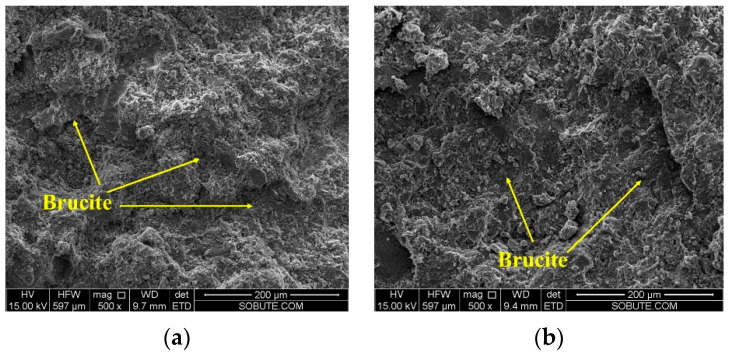
Morphologies of cement pastes with 4% MgO expansive agents: (**a**) 4% 65 s MgO and (**b**) 4% 120 s MgO.

**Figure 24:**
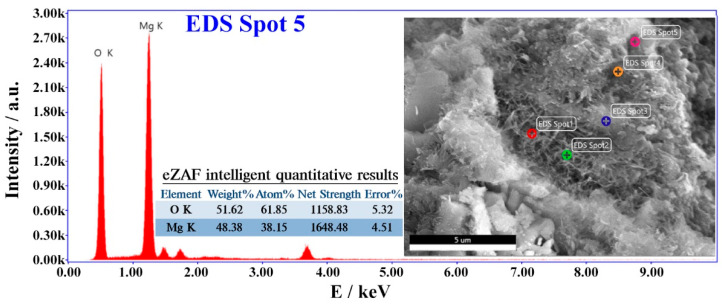
The energy spectrum test results of EDS Spot 5 in the microscopic picture of cement paste with 4% 120 s MgO expansive agent.

After being cured under variable temperature conditions, as shown in Figure 7, the morphologies of cement pastes with 4% MgO expansive agents were observed and are shown in Figure 23. No obvious microcracks were found in the cement paste with the 4% MgO expansive agent. MgO and hydrated Mg(OH)_2_ are not easy to observe under an electron microscope. The energy spectrum test results of EDS Spot 5 in the microscopic picture of the cement paste with the 4% 120 s MgO expansive agent are shown in Figure 24. At EDS Spot 5, Mg(OH)_2_ was formed.

**Figure 25:**
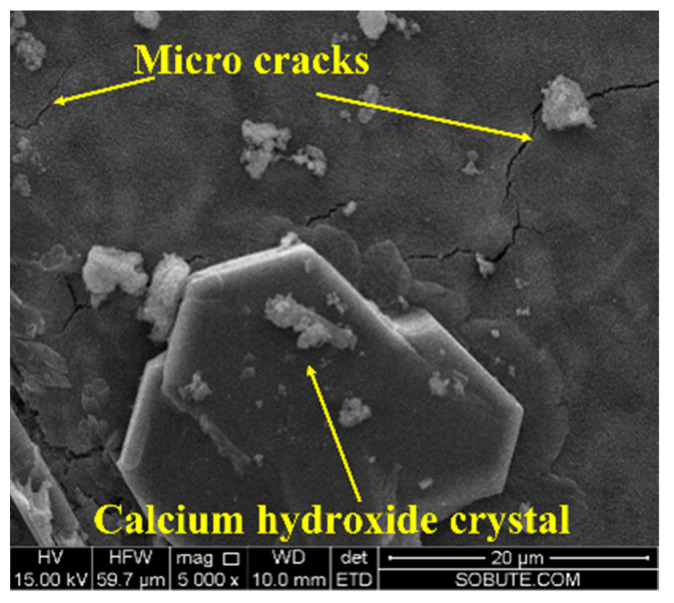
Morphology of cement paste with 6% CaO + 2% 220 s MgO expansive agent (×5000).

After being cured under variable temperature conditions, as shown in Figure 7, the morphology of the cement paste with the 6% CaO + 2% 220 s MgO expansive agent was observed and is shown in Figure 25. As can be seen from the figure, a large number of hexagonal plate Ca(OH)_2_ crystals appeared in the cement paste, and microcracks appeared in the cement paste with the 6% CaO and 2% 220 s MgO expansive agent.

The microscopic analysis shows that the CaO expansive agent can produce a large number of hexagonal plate Ca(OH)_2_ crystals in the cement paste. Microcracks appeared in the cement paste with the 6% CaO expansive agent. The formation of microcracks may be caused by the volume expansion of Ca(OH)_2_ generated by the hydration of the CaO expansive agent. In the cement paste with the 4% MgO expansive agent, no obvious microcracks were found, whereas microcracks appeared in the cement paste with the 6% CaO and 2% 220 s MgO expansive agent.

### 3.8. Discussion and Analysis

As mentioned above, different types of expansive agents have different hydration and expansion characteristics. In actual projects, the autogenous shrinkage and temperature-fall-induced shrinkage generated in C60 concrete in the early and middle hydration stages are large, with a wide temperature variation range. The large expansion of concrete should be compensated. In addition, the later autogenous shrinkage and temperature-fall-induced shrinkage of concrete are smaller, and microexpansion is needed to compensate for them, thus inhibiting their shrinkage to stabilize the expansion precompression stress formed in the early stage. Finally, shrinkage-free concrete should be achieved in all hydration stages.

The CaO expansive agent is beneficial to early expansion, while the MgO expansive agent can achieve middle- and late-age expansion; thus, a potentially good method to realize shrinkage-free concrete throughout the whole process is to design multicomponent expansive agents with CaO and MgO compounds.

In this study, the influence of the temperature history of core concrete in a steel tube arch on the expansion of C60 concrete with specific-activity MgO and CaO expansion components in certain proportions, a single CaO expansive agent, and a single MgO expansive agent in C60 concrete was investigated. The experimental results showed that the expansion effect of CaO expansive agents was predominantly reflected in the heating stage, while there was no expansion in the cooling stage. The reason for this is that CaO expansive agents completely reacted in the heating stage of this temperature history of C60 core concrete in a steel tube arch, according to microthermal experimental results. Moreover, the microscopic morphology showed that the expansion energy of the CaO expansive agent is larger. Microcracks appeared in the cement paste with the 6% CaO expansive agent. Liu et al. [45] found that the hydration degree of a CaO expansive agent is large at early ages, such that the hydration degree is 39.94% after being hydrated for 0.5 h at 10 °C in pure water, while the hydration degree is 52.47% after being hydrated for 5 min at 40 °C. Xia et al. [46] also stated that the hydration rate of the CaO expansive agent is fast in the early stages. The expansion deformation in the cooling stage was mainly caused by the MgO expansive agent. The 120 s MgO and 220 s MgO expansion agents resulted in continuous expansion during the cooling stage, and the expansion curve did not converge. The 220 s MgO expansive agent produced more expansion than 120 s MgO. The 65 s MgO expansive agent did not produce significant expansion deformation in the cooling phase. During the heating process of the concrete, the majority of 2% 65 s MgO reacted with water to form brucite, leading to the observed phenomenon. With the increase in the active reaction time of MgO, the hydration of MgO in the heating stage of concrete decreased, and the expansion of MgO in the cooling stage increased.

Following the actual temperature history of concrete, selecting the proper activity of MgO expansive agents and the ratio of CaO and MgO in multicomponent expansive agents could compensate for concrete shrinkage throughout the whole process. Zhao et al. [47] also found that a CaO and MgO composite expansive agent can compensate for concrete shrinkage not only during the early stage but also during the later stage at normal temperatures. Thus, the CaO and 220 s MgO composite expansive agent shows strong temperature sensitivity, which is suitable for compensating for the shrinkage of concrete in the case of a high-temperature rise and slow cooling rate. Furthermore, the CaO and 120 s MgO composite expansive agent is suitable for compensating for the shrinkage of concrete in the case of a low-temperature rise and fast cooling rate.

## 4. Conclusions

Utilizing appropriate expansive agents during cement hydration is one of the main techniques to compensate for concrete shrinkage and prevent voids and debonding between the steel pipe and core concrete in concrete-filled steel tubes. In this work, calcium oxide and magnesium oxide composite expansive agents were designed with different calcium/magnesium ratios and various types of MgO activities with different calcination temperatures and times. Their expansion and hydration properties in C60 concrete under variable temperature conditions were investigated, with the aim of simulating the real construction process. Subsequently, the effect of the calcium–magnesium ratio and magnesium oxide activity on deformation was analyzed. The main conclusions are summarized as follows:

(1) The expansion effects of 6% and 8% CaO expansive agents were predominantly reflected in the heating stage (from 20.0 °C to 72.0 °C at 3 °C/h), while there was no expansion in the cooling stage (from 72.0 °C to 30.0 °C at 3 °C/d, and then to 20.0 °C at 0.7 °C/h). The CaO expansive agent was hydrated in the early high-temperature heating stage, resulting in almost no expansion in the cooling stage. With the increase in the amount of the CaO expansive agent, the expansion deformation of concrete in the heating stage increased, and consequently, the internal expansion stress of concrete increased.

(2) The expansion deformation in the cooling stage was mainly caused by the MgO expansive agent. The 120 s MgO and 220 s MgO expansive agents resulted in continuous expansion during the cooling stage, and the expansion curve did not converge. The 220 s MgO expansive agent produced more expansion than 120 s MgO. During the heating process of the concrete, the majority of 2% 65 s MgO reacted with water to form brucite in large amounts, leading to its lower expansion deformation in the later cooling process. With the increase in the active reaction time of MgO, the hydration of MgO in the heating stage of concrete decreased, and the expansion of MgO in the cooling stage increased.

(3) The construction and building temperatures show a remarkable influence on the expansion performance of CaO and MgO composite expansive agents. In actual projects, the temperature history of concrete varies immensely from structure to structure on account of many factors, such as the material, environment, and structural dimensions. In accordance with the actual temperature history of concrete, selecting the proper activity of MgO and the ratio of CaO to MgO could compensate for concrete shrinkage throughout the whole process. The CaO and 220 s MgO composite expansive agent shows strong temperature sensitivity, which is suitable for compensating for the shrinkage of concrete in the case of a fast high-temperature rise and a slow cooling rate.

This work will guide the application of different types of CaO-MgO composite expansive agents in concrete-filled steel tube structures. More work should be conducted in the future to build a theoretical model of temperature effects on different components of the expansive agent to guide engineering projects.

## Figures and Tables

**Table 1:** Chemical compositions of cement, CaO expansive agent, and MgO expansive agents with different reaction times.

Raw Material	Reaction Times (s)	Chemical Composition (wt%)						
		MgO	CaO	SO_3_	SiO_2_	Al_2_O_3_	Fe_2_O_3_	Loss
Conch Cement	-	1.35	60.05	3.35	23.60	3.95	5.43	1.15
65 s MgO	65	90.17	1.87	-	1.83	1.39	2.15	2.59
120 s MgO	120	90.93	1.96	-	1.71	1.24	2.00	2.16
220 s MgO	220	91.38	2.24	-	2.36	1.32	1.12	1.58
CaO expansive agent	-	1.27	87.5	3.41	1.72	4.79	3.78	0.94

**Table 2:** Concrete mix designs of C60.

No.	W/C	Mix Ratio (kg/m^3^)							
		Cement	Fly Ash	CaO Expansive Agent	MgO Expansive Agent	Water	Sand	Small Basalt	Medium Basalt
C60-ref	0.29	416	104	0	0	151	720	249	746
6% CaO + 2% 65 s MgO	0.29	416	62.4	31.2	10.4	151	720	249	746
6% CaO + 2% 120 s MgO	0.29	416	62.4	31.2	10.4	151	720	249	746
6% CaO + 2% 220 s MgO	0.29	416	62.4	31.2	10.4	151	720	249	746
6% CaO + 4% 65 s MgO	0.29	416	52	31.2	20.8	151	720	249	746
6% CaO	0.29	416	72.8	31.2	0	151	720	249	746
8% CaO	0.29	416	62.4	41.6	0	151	720	249	746
4% 65 s MgO	0.29	416	83.2	0	20.8	151	720	249	746
4% 120 s MgO	0.29	416	83.2	0	20.8	151	720	249	746

**Table 3:** Hydration degree of MgO in cement paste under variable temperature conditions.

No.	6% CaO + 4% 65 s MgO	4% 65 s MgO	4% 120 s MgO
Hydration degree/%	87.3	85.5	70.3

## Data Availability

The datasets generated during and/or analyzed during the current study are available from the corresponding author upon reasonable request. All data generated or analyzed in this research are included in this published article. Additionally, readers can access all data used to support the conclusions of the current study from the corresponding author upon request.
